# Inpactor, Integrated and Parallel Analyzer and Classifier of LTR Retrotransposons and Its Application for Pineapple LTR Retrotransposons Diversity and Dynamics

**DOI:** 10.3390/biology7020032

**Published:** 2018-05-25

**Authors:** Simon Orozco-Arias, Juan Liu, Reinel Tabares-Soto, Diego Ceballos, Douglas Silva Domingues, Andréa Garavito, Ray Ming, Romain Guyot

**Affiliations:** 1 Department of Electronics and Automatization, Universidad Autónoma de Manizales, Manizales 170002, Colombia; simon.orozco.arias@gmail.com (S.O.-A.); rtabares@autonoma.edu.co (R.T.-S.); 2 FAFU and UIUC-SIB Joint Center for Genomics and Biotechnology, Fujian Agriculture and Forestry University, Fuzhou 350002, China; relaxljliu@sina.com (J.L.); rming@life.uiuc.edu (R.M.); 3 Department of Systems and Informatics, Universidad de Caldas, Manizales 170002, Colombia; diego.ceballos@ucaldas.edu.co; 4 Department of Botany, Instituto de Biociências, Universidade Estadual Paulista, UNESP, Rio Claro, SP 13506-900, Brazil; doug@rc.unesp.br; 5 Department of Biological Sciences, Universidad de Caldas, Manizales 170002, Colombia; neagef@gmail.com; 6 Department of Plant Biology, University of Illinois at Urbana-Champaign, Champaign, IL 61801, USA; 7 Institut de Recherche pour le Développement (IRD), CIRAD, Université de Montpellier, Montpellier 34394, France

**Keywords:** Inpactor, transposable elements, LTR retrotransposons, parallel programming, pineapple, HPC

## Abstract

One particular class of Transposable Elements (TEs), called Long Terminal Repeats (LTRs), retrotransposons, comprises the most abundant mobile elements in plant genomes. Their copy number can vary from several hundreds to up to a few million copies per genome, deeply affecting genome organization and function. The detailed classification of LTR retrotransposons is an essential step to precisely understand their effect at the genome level, but remains challenging in large-sized genomes, requiring the use of optimized bioinformatics tools that can take advantage of supercomputers. Here, we propose a new tool: Inpactor, a parallel and scalable pipeline designed to classify LTR retrotransposons, to identify autonomous and non-autonomous elements, to perform RT-based phylogenetic trees and to analyze their insertion times using High Performance Computing (HPC) techniques. Inpactor was tested on the classification and annotation of LTR retrotransposons in pineapple, a recently-sequenced genome. The pineapple genome assembly comprises 44% of transposable elements, of which 23% were classified as LTR retrotransposons. Exceptionally, 16.4% of the pineapple genome assembly corresponded to only one lineage of the *Gypsy* superfamily: *Del*, suggesting that this particular lineage has undergone a significant increase in its copy numbers. As demonstrated for the pineapple genome, Inpactor provides comprehensive data of LTR retrotransposons’ classification and dynamics, allowing a fine understanding of their contribution to genome structure and evolution. Inpactor is available at https://github.com/simonorozcoarias/Inpactor.

## 1. Introduction

Transposable Elements (TEs) constitute the main part of the nuclear DNA content of plant genomes. This is particularly true for large genomes of cereals such as wheat, barley and maize, for which up to 85% of the sequenced DNA is classified into repeated sequences [1]. On the contrary, compact genomes such as those of *Arabidopsis thaliana* (10%) and the carnivorous plant *Utricularia gibba* (3%) show a lesser content of TEs [2], suggesting that their copy numbers may vary drastically and are associated with genome size variation in plant genomes [3]. Occasionally, a rapid increase in copy numbers of a few TE families may lead colossal genome size variations between related species [4]. TEs can be activated through a large panel of biotic and abiotic stresses ([5,6]), suggesting that they could play a significant role in the environmental adaptation of species [7].

Transposable elements are traditionally classified according to their mechanism of transposition [8]: Class I or retrotransposons move through an RNA intermediate via a “copy and paste” mechanism, and Class II or DNA transposons do not use an RNA intermediate and move via a “cut and paste” mechanism. Class I includes LTR (Long Terminal Repeats) retrotransposons and non-LTR retrotransposons, such as LINEs (Long Interspersed Nuclear Elements) and SINEs (Short Interspersed Nuclear Elements), while Class II contains Terminal Inverted Repeat (TIR) DNA transposons and Helitrons. The most common transposable elements in plants genomes are LTR retrotransposons, because they replicate by a “copy and paste” mechanism. They represent 75% of the maize genome [9], 67% of wheat ([1,10]), 55% of *Sorghum bicolor* [11] and 42% of the coffee genome [12]. The sequences of full-length LTR retrotransposons usually carry two coding genes: the GAG gene involved in TE packaging into a virus-like particle and the Pol gene coding for the enzymatic machinery mainly involved in the retro-transcription of the element. LTR retrotransposons are further classified into *Gypsy* and *Copia* super-families according to the position of the integrase domain in the Pol coding region, and they are further separated into lineages and families according to their structural features and domain similarities [8]. Six domains are particularly important for the mobility of the elements. The GAG (Group Specific Antigen) domain is involved in the formation of virus-like particles; the Aspartic Protease (AP) is responsible for processing the polyprotein of the element into smaller proteins; the Reverse Transcriptase domain (RT) is the key enzyme involved in DNA synthesis (using an RNA template); the RNase H domain degrades the RNA template in the DNA-RNA molecule; while the Integrase domain (Int) catalyzes the insertion of the retrotransposon cDNA into the host genome. Occasionally, an Envelope (Env)-like domain is present [8]. In angiosperms, the main *Gypsy* lineages are the closely-related *TAT* and *Athila* lineages, and the *Galadriel*, *Reina*, *CRM* (Centromeric Retrotransposon in Maize) and *Del* lineages [13]. The main *Copia* lineages are classified as *Tork*, *Retrofit*, *Oryco* and *SIRE*. The *Bianca* lineage was also recently described as part of the *Copia* super-family ([14,15]).

Defective elements, lacking several or all of these domains involved in mobility, can be classified as non-autonomous LTR retrotransposons (LTR-RT) elements. They are further sub-classified into TRIM (Terminal-repeat Retrotransposon In Miniature) [16], LARD (Large Retrotransposon Derivative) [17], BARE-2 (Barley RetroElement-2) [18] and TR-GAG (Terminal repeat with Gag domain) [19], according to their internal structures.

Since TEs correspond to the major part of plant genomes, their precise and exhaustive annotation, particularly in large genomes, remains a difficult and extensive work. More efforts in identification and annotation are necessary in partial draft genomes or in the case of new or highly degenerated repeated elements. In the last few years, several tools allowing one to identify and annotate transposable elements based on their structure and/or similarity were developed ([20,21]). REPET, one of these tools, has been developed to identify and classify transposable elements at a whole genome sequence scale. It was recently used to annotate TEs in several plant genomes, such as wheat [1], *Solanum pennellii* (a wild relative of tomato; [22]), *Coffea canephora* [12] and *Capsella rubella* [23].

However, available tools for the classification of TEs, and more particularly LTR-retrotransposons, such as TEclass [24], Repclass [25], Pastec [26], LTRsift [27] and LTRclassifier [28], provide limited information about the identification of super-families, while none of them are capable of classifying elements into lineages, nor identifying non-autonomous elements. Furthermore, optimized bioinformatics tools taking advantage of current supercomputers are now necessary to analyze and classify the large set of genomes and transposable elements available.

Computational approaches such as supercomputing, artificial intelligence [29] and data mining [30] are currently used for biological sciences, including sequences comparisons, nucleic acid secondary structure prediction and molecular dynamics [31], demonstrating the importance of speeding-up the analysis processes for large genomes [32]. Message Passing Interface (MPI) is a standard library for parallel programming [33], which is able to take advantage of multi-cores (like servers with many CPUs), many-cores (like GPUs) or heterogeneous (interaction between CPUs and GPUs [34]) architectures. MPI is capable of running in parallel many sub-problems that are previously divided given three focuses: (i) executing independent processes simultaneously; (ii) decomposing the main problem into tasks and resolving them in parallel; and (iii) introducing parallelism at instruction levels [35].

Pineapple (*Ananas comosus* L. 2n = 2x = 50) is a species indigenous to South America, belonging to the family Bromeliaceae (order Poales). Pineapple is the second most important tropical fruit crop after mango (FAO, http://www.fao.org/) and the most economically important species in the family Bromeliaceae. It is also the most economically important crop that assimilates carbon using Crassulacean Acid Metabolism (CAM) and is consequently a model to study the CAM photosynthesis pathway [36]. Over many years, genetic and genomic resources have been developed and reported for pineapple, including genetic maps with F1 and F2 populations [37] and expressed Sequence Tags (EST) and transcriptomes [38]. Only one previous study reported the presence of LTR-retrotransposon *Del* elements [39] in the pineapple genome. Now, the release of the pineapple genome sequence [40], with a draft that covers 72% of the estimated 526-Mb genome, offers the possibility of a large-scale analysis of the TE content.

In this study, we report the development of Inpactor, a parallel and scalable pipeline, able to classify LTR retrotransposons, to identify autonomous and non-autonomous elements, to perform RT-based phylogenetic trees and to analyze their insertion times using High Performance Computing (HPC) techniques. Inpactor was tested through a comprehensive analysis based on the identification and annotation of transposable elements in the pineapple genome. The pineapple genome assembly is comprised of 44% of transposable elements, of which 23% were classified as LTR retrotransposons and 9% as non-autonomous LTR retrotransposons. Only one lineage of the *Gypsy* superfamily: *Del*, corresponds to 16.4% of the pineapple genome assembly, suggesting that this lineage has undergone a significant increase in its copy numbers. Most full-length LTR retrotransposons were recently inserted (<2 Mya), reinforcing the hypothesis that they represent one of the most dynamic fractions of the pineapple genome. Inpactor provides comprehensive data of LTR retrotransposons’ classification and dynamics at the lineage level, allowing a fine understanding of their contribution to genome structure and evolution.

## 2. Materials and Methods

### 2.1. Implementation of Inpactor

**Figure 1:**
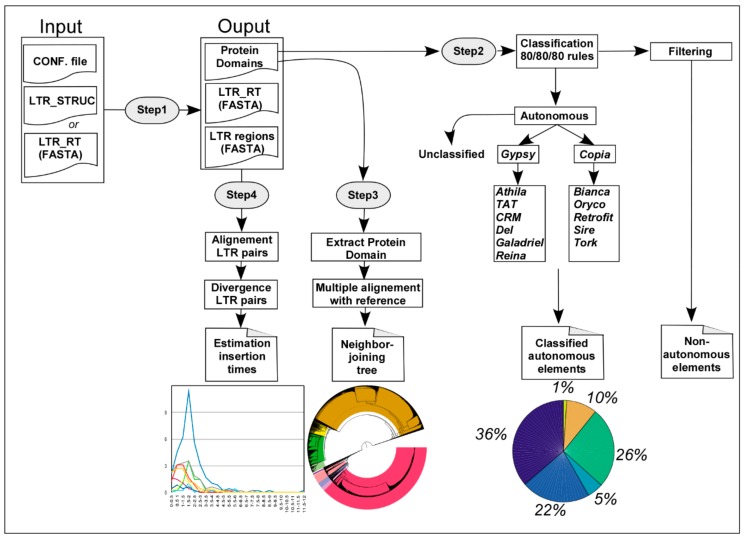
Representation of the different steps of the Inpactor pipeline.

Inpactor is composed of four modules (Figure 1) and was developed using the Message Passing Interface (MPI) standard, in C language. It requires input parameters to be declared in a configuration file (Supplementary S1), where it is possible to define general information such as input file types (LTR_STRUC [41] output, REPET’s TEdenovo output in FASTA format or a genome FASTA file), result directory, verbose mode and clean mode at the end of the execution. In addition, each module requires that different parameters be indicated in the configuration file.

Step 1, preprocessing module. The objective is to sort information and features from the LTR_STRUC output or the FASTA files submitted to Inpactor, such as the full element sequence, LTR identity, length and sequences using tools from EMBOSS [42] and BLASTX results against six references domains (GAG, RT, INT, RNAse H, AP and ENV) available at the Gypsy Database Project [15].

Step 2, classification module. It performs a classification using results from Step 1 as follows: (i) if the element carries at least one of the following domains: RT, INT, and RNAse H, with keywords RLC or RLG, the LTR-RT is classified as a putative autonomous-family element (*Copia* or *Gypsy*); (ii) if the element does not carry any domain, it is classified as a non-autonomous element (TRIM if the LTR-RT length is lower than 2000 bases and LARD if the LTR-RT length is greater than or equal to 2000 bases); (iii) if the element has only a GAG domain or a GAG and an AP domain, the element falls into the non-autonomous TR-GAG elements. Non-autonomous elements are not reclassified into super-families or lineages with autonomous elements. Elements with domains from both super-families (*Copia* and *Gypsy*) are considered as unclassified (possible chimeric elements). In addition, Inpactor creates an extra text file, which contains all LTR-RTs that are unclassified and thus named “no-class elements”. Finally, the complete sequence of each classified and unclassified LTR retrotransposon (Figure 1) is extracted. A re-classification is performed following the 80-80-80 rule from Wicker et al. [8] for unclassified elements (Step 2, classification module parameters). Unclassified LTR-RT elements are re-analyzed with previously classified elements by similarity using Censor [43]. If the alignment covers a minimum of 80% of the unclassified element, with a minimum of 80% of nucleotide identity, and a minimum of 80 bases aligned (the 80/80/80 rule), the unclassified element is re-classified into the reference element [8].

Step 3, domain extraction module. Reverse Transcriptase (RT) domain sequences are extracted from each autonomous-family element, because this domain is the most conserved and appropriate for phylogenetic analysis (Figure 1). Other domains from the LTR-RT polyprotein might be used alternatively. BLASTX is executed using the FASTA file of all autonomous-family elements as the query and the reference RT domain database (the Gypsy Database Project [15]). Sequences that match with the database are extracted (extractseq, EMBOSS), and the domain is translated into amino acids using Genewise [44] with the option −pep. Only translated sequences larger than 200 amino acids are conserved for further analysis.

Step 4, LTR-RT insertion times’ analysis and phylogenetic analysis. Using the FASTA protein file from the RT domain extraction module, a multiple alignment is performed using Mafft [45] with the option −thread to indicate the number of cores. Then, a phylogenetic tree is created based on the maximum likelihood method with Mafft using −retree and −treeout options with the multiple alignment obtained previously (Figure 1). The insertion times of full-length copies, as defined by a minimum of 80% of nucleotide identity over 100% of the reference element length, are dated [19]. Timing of insertion is based on the divergence of the 5′ and 3′-LTR sequences of each copy. The two LTRs are aligned using Stretcher (EMBOSS) and the divergence calculated using the Kimura 2-parameter method implemented in Distmat (EMBOSS) [46]. The insertion dates are estimated using an average base substitution rate of 1.3 × 10^−8^ as the default parameter [47]. This default parameter can be changed in the configuration file.

Inpactor produces different types of files: a sequence file (FASTA), a global alignment matrix, a phylogenetic tree and tabular files with the insertion time analysis. In addition, the Preprocessing module produces one tabular file, which contains all information from the LTR_STRUC output; the Classification module creates one tabular file and one FASTA file for each LTR-RT type found, including the unclassified. Finally, the domain extraction section generates only one FASTA file with all of the domains found.

Inpactor requires using external bioinformatics software to perform specific functions such as sequences extraction and translation: NCBI-Blast (v.2.5.0, https://www.ncbi.nlm.nih.gov/BLAST/), EMBOSS (v.6.6.0, http://emboss.sourceforge.net), Wise 2 (v.2.4.0, http://www.ebi.ac.uk/~birney/wise2/), OpenMPI (v.1.8.8, https://www.open-mpi.org/), Censor (v.4.2.29, http://www.girinst.org/downloads/software/censor/), Mafft (v.7.305, http://mafft.cbrc.jp/alignment/software/) and LTR_FINDER (v.1.0.5, https://code.google.com/archive/p/ltr-finder/).

### 2.2. Availability of Inpactor

Inpactor’s source code can be found at https://github.com/simonorozcoarias/Inpactor, under the GNU GPLv3 license and is composed of one source code in C language, two bash scripts and an example of the configuration file. All of these need to be in the same folder. Installation instructions and a user manual are available at https://github.com/simonorozcoarias/Inpactor/blob/master/User%20manual%20Inpactor%20V%201.0%20final.pdf. Sample data and results are also available.

### 2.3. Computational Resources

All executions were done using a server with a 32-core Xeon E5-2670 (with HT enabled), 256 GB of RAM and the Centos 6.7 operating system, managed by Slurm [48]. All software used by Inpactor were installed in a non-standard directory and were loaded using Environmental Modules [49].

### 2.4. Sequence Data Sources

Inpactor was tested using five plant genomes with different genome sizes. *Arabidopsis thaliana* (117 Mb, http://plants.ensembl.org/Arabidopsis_thaliana/Info/Index) and maize (*Zea mays*, 2048 Mb; http://plants.ensembl.org/Zea_mays/Info/Index) were downloaded from the Ensembl genomes project [49]; rice (*Oryza sativa*, 362 Mb; http://ensembl.gramene.org/Oryza_sativa/Info/Index) was downloaded from the Gramene Project [50]; and Robusta coffee (*Coffea canephora*, 553 Mb; http://coffee-genome.org/) was downloaded from Coffee Genome Hub Project [51]. The pineapple genome sequence (variety “F153”) was generated from a combination of Illumina, Moleculo, PacBio, and 9400 Bacterial Artificial Chromosomes (BACs) and released [40]. The genome sequences were deposited at the iPlant CoGe database, and they can be downloaded at https://genomevolution.org/CoGe/NotebookView.pl?nid=937. The final assembly includes 382 Mb, corresponding to 72.6% of the estimated 526-Mb genome size.

### 2.5. Identification of Repeated Elements

REPET (TEdenovo package V.2.2-RC) [52] was used to find and classify repeated sequences in the pineapple genome sequences. In total, 3380 scaffolds accounting for 382,063,720 bp were processed. Consensus sequences obtained in REPET were annotated according to the REPBASE database (v.19.6, http://www.girinst.org/repbase/). They were named according to the acronym classification developed by Wicker and coworkers [8] (i.e., DHX (Helitron), DMX (Maverick), DTX (TIR Transposon), DXX (MITE) for Class II elements and RIX (LINE), RLX (LTR retrotransposon), RSX (SINE), RXX (unclassified or non-autonomous retrotransposons), RYX (DIRS) for Class I elements). Consensus sequences were classified as chimeric if they showed characteristics of more than one classification, representing potential nested elements. Additional tools were used to specifically predict full-length LTR retrotransposons (LTR_STRUC, [41]) based on their structure in order to complete the REPET detection.

### 2.6. Annotation, Phylogenetic Analysis and Insertion Time Analysis of LTR Retrotransposons

Consensus sequences from REPET that were identified, as “complete” (autonomous) or “incomplete” (non-autonomous) LTR retrotransposons were further classified into lineages and families using Inpactor. At the genome level, putative RT domains were identified using BLASTX [53], with an e-value cut-off of 1 × 10^−4^, and translated into amino acid sequences using Genewise [44]. The resulting RT sequences (with a minimum length of 150 residues) and reference RT domains from the Gypsy Database 2.0 were aligned, and a maximum likelihood tree was inferred and edited with Figtree (http://tree.bio.ed.ac.uk/software/figtree/). Insertion time analysis of LTR retrotransposons was performed as in Dupeyron et al., 2017, with the average substitution rate of 1.3 × 10^−8^ as implemented in Inpactor. LTR retrotransposons were used to annotate pineapple pseudo-molecules using RepeatMasker (-div 20 option; [54]; http://www.repeatmasker.org).

## 3. Results and Discussion

### 3.1. Testing Inpactor on Reference Plant Genomes

Genome annotation studies require the annotation and detailed classification of transposable elements, and more particularly LTR retrotransposons, representing the main part of plant genomes. Classification into main classes and lineages, insertion time analysis and phylogenetic analysis [55,56] constitute basic information for understanding the impact, dynamics and evolution of LTR retrotransposons. Inpactor has been developed to combine automatic annotation, classification, insertion time and phylogenetic analyses into a limited time process, taking advantage of supercomputers.

We first tested Inpactor using several numbers of cores (1, 4, 8, 16 and 32), with 10 repetitions for each experiment in order to calculate the speed-up and average run time per module (Table 1 and Supplementary S2).

**Figure 2:**
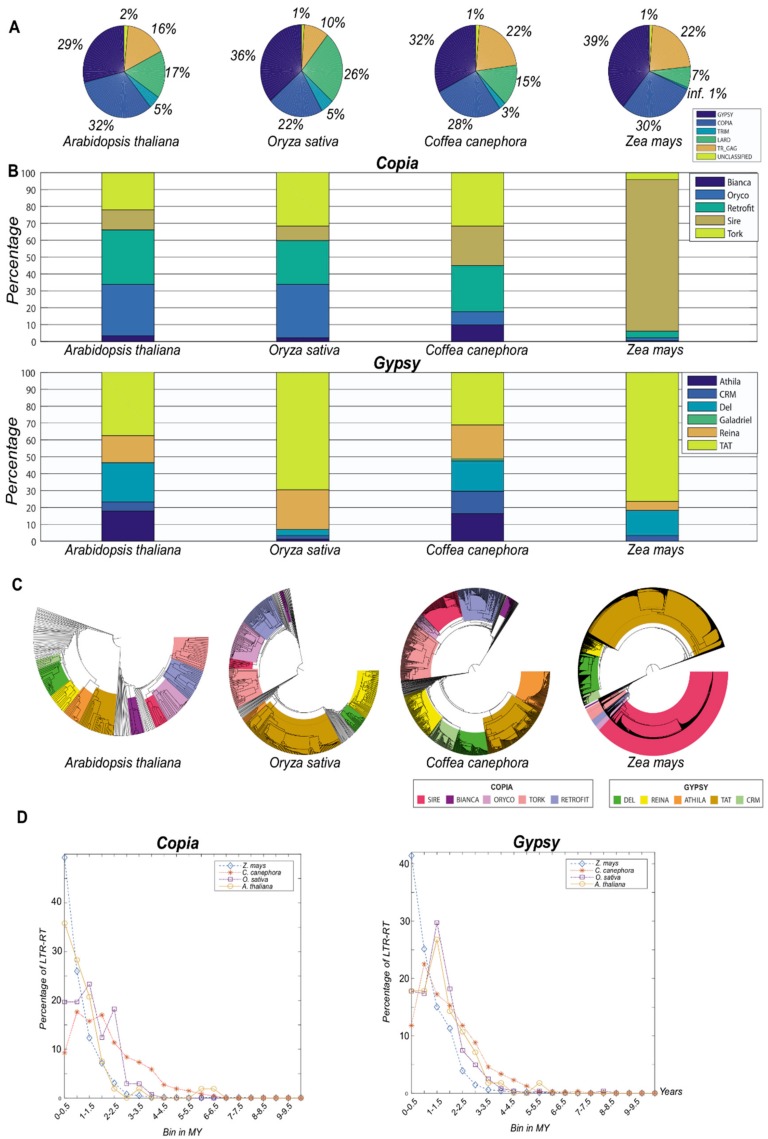
Inpactor results for the four species tested (*Arabidopsis thaliana*, *Oryza sativa*, *Coffea canephora* and *Zea mays*) based on LTR_STRUC detection. (**A**) Initial classification of LTR-RTs into autonomous (*Gypsy* and *Copia*) or non-autonomous (Terminal-repeat Retrotransposon In Miniature (TRIM), Large Retrotransposon Derivative (LARD) or Terminal repeat with Gag domain (TR-GAG)); (**B**) classification of the autonomous elements into lineages showing the variability that can be found in plant genomes; (**C**) phylogenetic trees using the RT domain; (**D**) insertion time analysis using autonomous elements (*Copia* and *Gypsy*).

Four different plant genomes (*Arabidopsis thaliana*, *Oryza sativa*, *Coffea canephora* and *Zea mays*) were used. Only the 32 cores’ outputs were used for classifying predicted LTR-RTs into autonomous elements (*Gypsy* and *Copia* super-families and lineages) and for filtering putative non-autonomous element types (LARD, TRIM and TR_GAG; Figure 2 and Supplementary S3). Autonomous elements were sub-classified into lineages, and a phylogenetic tree per genome was constructed using the output files (Figure 2). Inpactor provided the insertion time analyses, indicating the insertion activity of LTR-RT elements over recent periods of time (Figure 2 and Supplementary S4).

**Figure 3:**
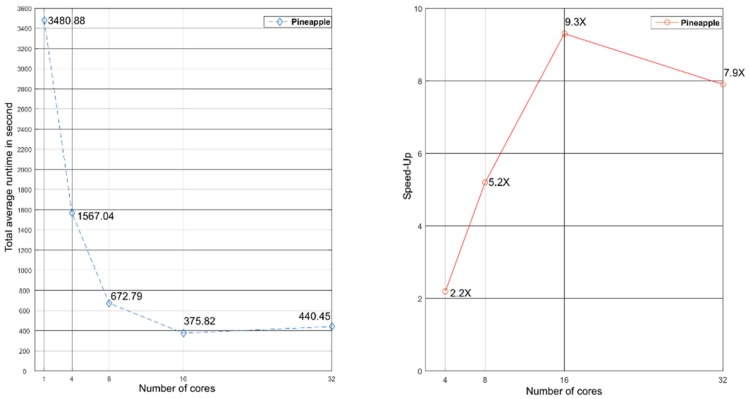
Total average runtime and speed-up of Inpactor using 4, 8, 16 and 32 cores with pineapple data.

Executions were performed using one server (Supplementary S5–S8), with the 80-80-80 rule option disabled. Each Inpactor module was executed independently in the correct order (i.e., preprocessing, classification, domain extraction, insertion time and phylogenetic tree creation) to calculate the runtime of each module. The total runtime is the sum of each runtime module. Finally, Inpactor was run on the pineapple genome sequence similarly to the four reference genomes used in order to study its LTR retrotransposons diversity (Figure 3 and Supplementary S9).

Inpactor can use different input files such as the LTR_STRUC output files and any FASTA files from other predictors of full-length elements LTR retrotransposons. LTR_STRUC is a relatively slow algorithm (running under a Windows-XP PC), compared to more recent prediction software [57], but it seems to offer a low percentage of false positives in plant genomes and an overall low number of putative elements. In the future, Inpactor will integrate more recent software used to predict LTR retrotransposons, such as LTRharvest [57], LTR-FINDER [58], and LTR_retriever [59]. Additionally, we will also include Hidden Markov Models (HMM) to perform a more sensitive annotation of protein domains. Inpactor uses a Shell script gluing together other programs to construct analysis. To speed up the overall analysis, Inpactor will be implemented as a single C binary.

As expected, Inpactor’s results showed a different composition of LTR retrotransposon lineages in the reference genomes based on the detection of LTR_STRUC’s full-length elements. Our results illustrate considerable variation in the classification of elements, despite that the quantitative detection of elements may be biased by the quality of the genome sequence and assembly. Insertion time and phylogenetic tree modules also provided evidence of different insertional activity of LTR retrotransposons during similar periods of time.

Inpactor surpasses other classification tools for LTR retrotransposons such as TEclass, Pastec and LTR classifier. None of them are able to give detailed information about the LTR retrotransposons’ lineages, to identify non-autonomous elements and to estimate insertion times. As a consequence, it was not possible to compare the performance of Inpactor with these tools. Similarly, it was not possible to compare the classification of Inpactor with those from the published genomes of *A. thaliana*, rice, coffee and maize due to the lack of detailed information.

### 3.2. Using Inpactor on the Pineapple Genome

**Figure 4:**
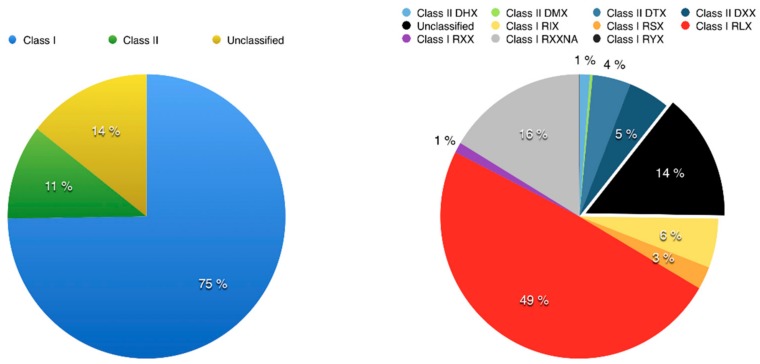
Transposable element abundance found in the pineapple genome. Classification of Transposable Elements (TEs) (left) and a detailed composition using the acronym classification developed by Wicker [8] (right) are presented: DHX (Helitron), DMX (Maverick), DTX (TIR Transposon), DXX (MITE), RIX (LINE), RLX (LTR retrotransposon), RSX (SINE), RXX (unclassified or non-autonomous retrotransposons), RYX (DIRS).

**Figure 5:**
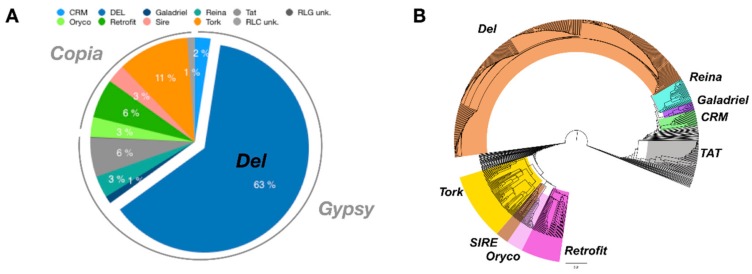
LTR retrotransposon lineages identified in consensus sequences identified by Inpactor. (**A**) Proportion of the different lineages in consensus sequences. The *Del* lineage represented 64% of all LTR retrotransposon annotated consensus sequences. (**B**) Phylogenetic analysis of annotated LTR retrotransposon consensus sequences.

To use Inpactor on the pineapple genome, we first identified repeated sequences with the REPET TEdenovo package. After clustering and cleaning, 2860 consensus sequences were obtained from the genomic scaffolds and classified according to their structural features and similarities with the REPBASE protein database [60]. As a result, 75% of them were classified into Class I elements (retrotransposons) and 11% into Class II (DNA transposons), following the hierarchical classification proposed by Wicker and coworkers [8] (Supplementary S10). The remaining 14% of repeats were not identified at this step (Figure 4). Furthermore, 1402 LTR retrotransposon consensus sequences (RLX) were identified via TEdenovo, but 1148 sequences were classified as incomplete elements due to missing structural features detected by REPET [52]. Among the 1402 LTR retrotransposon consensus, 939 RLX consensus sequences were annotated and classified into lineages using Inpactor. Most of them (714, 76%) fell into the *Gypsy* superfamily, and more particularly into the *Del* lineage (590, 63%, Figure 5A). Most consensus elements that were classified into the *Del* lineage were closely related to the *Peabody* family based on their RT domains. We did not identify any reverse transcriptase domains from the *Athila* and *Bianca* lineages ([61,62]). Only 225 consensus sequences (23%) belonged to the *Copia* super-family. The remaining LTR retrotransposon consensus sequences that did not carry any recognizable RT domain were classified by Inpactor as TR-GAG (353) or other non-autonomous elements (RXX, 97); probably built from deletion derivative elements. In total, Inpactor did not classify 13 consensus sequences. Finally, Inpactor recovered RT domains for each consensus and released a maximum likelihood phylogenetic tree (Figure 5B), confirming the classification and the overrepresentation of consensus sequences from the *Del* lineage.

**Figure 6:**
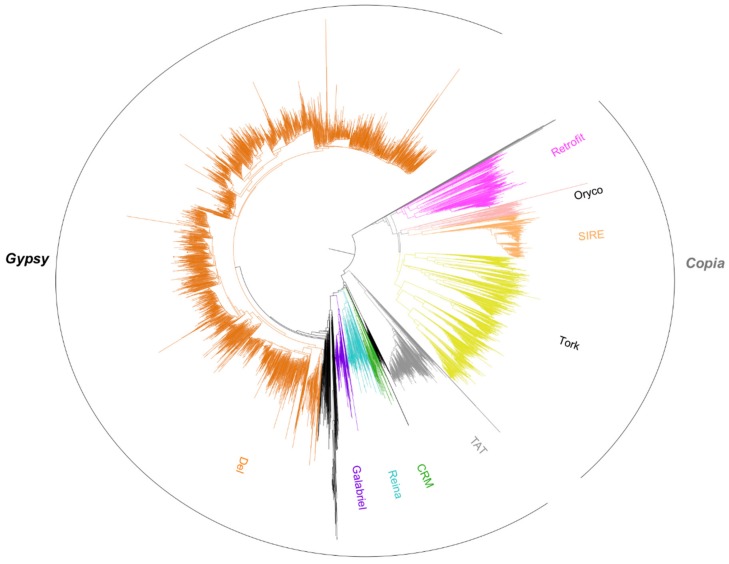
Phylogenetic analysis of 6379 Reverse Transcriptase (RT) domains from the pineapple genome assembly. RT domains were classified into *Gypsy* and *Copia* super-families and lineages using the reference RT domain from the Gypsy Database. The branches from the Gypsy *Del* family are represented in orange.

RT domains were also directly recovered from the pineapple genome sequence, and 6379 aligned amino acid sequences were used to construct a phylogenetic tree to classify *Gypsy* and *Copia* super-families and lineages at the genome level (Figure 6). Similarly to the consensus analysis, the phylogenetic tree indicates the overrepresentation of RT domains from the *Del* lineage at the genome level. Most of the branches were closely linked to the *Peabody* family RT domain, confirming previous observations at the molecular level [40].

### 3.3. Pineapple LTR Retrotransposons Abundance and Dynamics

The final LTR-retrotransposon repertoire annotated by Inpactor, composed of 1389 sequences (for 5,263,860 bp of sequence), was used for pineapple pseudochromosome annotation using RepeatMasker. This repertoire masked 31.08 percent (118,709,778 bp) of the genome sequence. *Copia* and *Gypsy* elements represent 2.7% and 19.3%, respectively, of the genome, while non-autonomous elements represent 1.4% for RXX and 7.7% for TR_GAG. Indeed, the *Del* lineage represents a significant proportion of the genome with 16.4%. Along with pseudo-molecules, LTR retrotransposons range from 22.16 (LG4) to 33.18% (LG24), with the notable exception of the LG25 pseudo-molecule showing an overall percentage of 10.70% (Supplementary S11 and S12). *Del*, the most abundant lineage, ranges from 11.48 (LG17) to 18.65% (LG24), along with pseudo-molecules. Once again, the pseudo-molecule LG25 showed the lowest percentage of *Del* with 4.74%. The very low detection of LTR retrotransposons on LG25 remains intriguing and might be a result of reduced pericentromeric regions. Indeed, this reduction could also originate from difficulties in assembling reads from highly repetitive regions. Beside *Del*, non-autonomous elements (TR-GAG) represent the most significant group with a variation between 5.79% and 7.78%.

**Figure 7:**
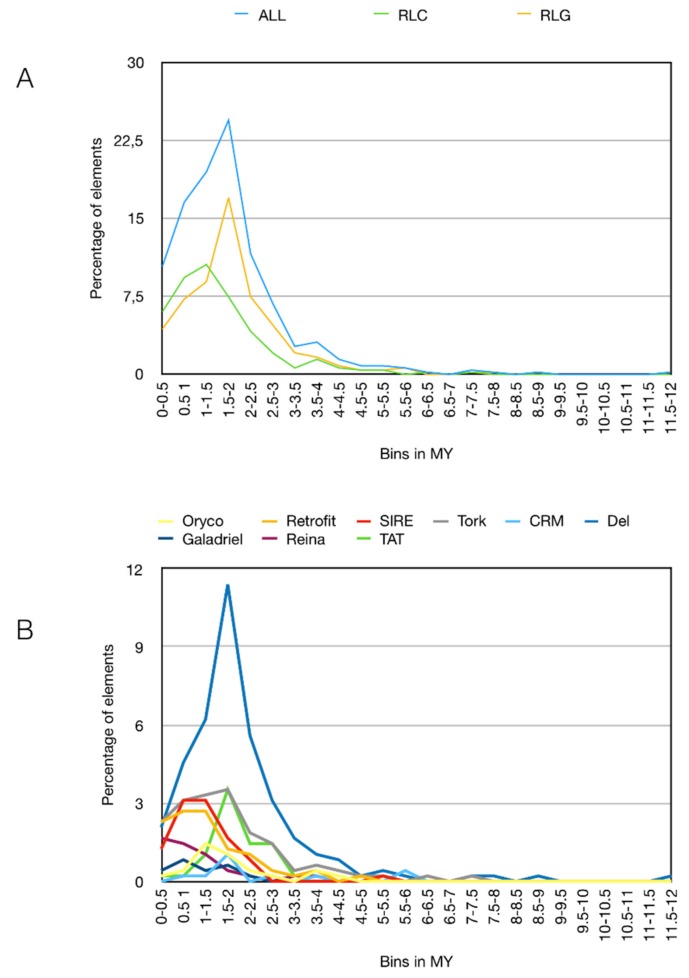
Timing of full-length LTR retrotransposon insertions. (**A**) Blue, yellow and green lines represent respectively the percentage of *Gypsy* and *Copia* full-length LTR retrotransposons per bins of 0.5 Million Years (MY); (**B**) colored lines represent the percentage of full-length LTR retrotransposon lineages per bins of 0.5 MY. Only the full-length LTR retrotransposons found by LTR_STRUC were used here. An average base substitution rate of 1.3 × 10^−8^ was used as the default [47].

The pineapple genome was also processed by LTR_STRUC, and the output was used to estimate the time insertion of full-length LTR retrotransposons by Inpactor (Figure 7). Two different peaks were observed at 1.5–2 Million Years (MY) for *Gypsy* elements and at 1–1.5 MY for *Copia*, suggesting two different rounds of LTR retrotransposon amplification. The time insertion analyzed by lineages confirmed the amplification of *Del* lineages at 1.5–2 MY as the origin of its large copy numbers in the pineapple genome (Figure 7B).

## 4. Conclusions

In conclusion, Inpactor is a unique tool providing an exhaustive classification and analysis of LTR retrotransposons. It performs the classification of elements into super-families and lineages and efficiently filters non-autonomous elements. An additional benefit of Inpactor is the availability of an RT-based phylogenetic tree for supporting classification into lineages and a lineage-based time insertion analysis for analyzing elements’ dynamics. Finally, the analysis of the pineapple genome with Inpactor provided fast and interesting information about the abundance and dynamics of LTR retrotransposons. It also suggests the good complementarity of REPET and Inpactor for the efficient and rapid classification and analysis of LTR retrotransposons.

## Figures and Tables

**Table 1:** Results of Inpactor on 4 different plant genomes.

Species	Total Average Sequential Runtime in Seconds	Sequential Standard Deviation in Seconds	Total Average Parallel Runtime in Seconds	Parallel Standard Deviation in Seconds	Number of Cores	Speed-Up
*Arabidopsis thaliana*	995.3	14.42	361.28	5.82	4	2.8
			201.16	12.65	8	4.9
			134.5	9.24	16	7.4
			158.47	11.28	32	6.3
*Oryza sativa*	3228.7	94.07	1099.67	42.48	4	2.9
			677.25	41.65	8	4.8
			428.02	24.64	16	7.5
			412.75	18.61	32	7.8
*Coffea canephora*	9569.48	11.91	3292.15	155.64	4	2.9
			2029.39	108.92	8	4.7
			1143.97	23.64	16	8.4
			1015.44	31.71	32	9.4
*Zea mays*	65,031.07	1143.79	22,186.47	306.43	4	2.9
			11,657.74	582.24	8	5.6
			8452.74	394.94	16	7.7
			7907.58	495.85	32	8.2

## Supplementary Materials

The following are available online at http://www.mdpi.com/2079-7737/7/2/32/s1, Supplementary S1: Example of the configuration file necessary to run Inpactor, Supplementary S2: Runtimes, standard deviations and break-down runtimes for *Arabidopsis thaliana*, *Oryza sativa*, *Coffea canephora* and *Zea mays* genomes, Supplementary S3: Classification of LTR-RTs found by Inpactor in *Arabidopsis thaliana*, *Oryza sativa*, *Coffea canephora* and *Zea mays*, Supplementary S4: Insertion time analysis done by Inpactor of autonomous elements (*Copia* and *Gypsy*) using *Arabidopsis thaliana*, *Oryza sativa*, *Coffea canephora* and *Zea mays*, Supplementary S5: Total average runtime of Inpactor using *Arabidopsis thaliana*, *Oryza sativa*, *Coffea canephora* and *Zea mays*, Supplementary S6: Comparison between break-down runtimes for each Inpactor module using one and 32 cores with the *Zea mays* genome, Supplementary S7: Speed-up of Inpactor using 4, 8, 16 and 32 cores and the *Arabidopsis thaliana*, *Oryza sativa*, *Coffea canephora* and *Zea mays* genomes, Supplementary S8: Average runtime of each Inpactor module using the *Zea mays* genome, Supplementary S9: Comparison between break-down runtimes of each Inpactor module using one and 32 cores with the Pineapple genome sequences, Supplementary S10: Number and classification of TE consensus found by TEdenovo in the Pineapple sequence, Supplementary S11 and 12: Percentage/proportion of the different LTR retrotransposon lineages and non-autonomous types for each pineapple pseudochromosome.
