# Supplementary material for: Inpactor, Integrated and Parallel Analyzer and Classifier of LTR Retrotransposons and Its Application for Pineapple LTR Retrotransposons Diversity and Dynamics
Source: Biology (Basel). 2018 May 25;7(2):32. doi: 10.3390/biology7020032 (PMC6022998; doi:10.3390/biology7020032)
Supplement: Supplementary file 1 [file biology-07-00032-s001.zip › Supplementary S10.docx]

**Supplementary S10. Number and classification of TE consensus found by TEdenovo in the Pineapple sequence.**

The classification acronyms are fully described in (Wicker, Sabot et al. 2007). Class I : RIX LINE, RL
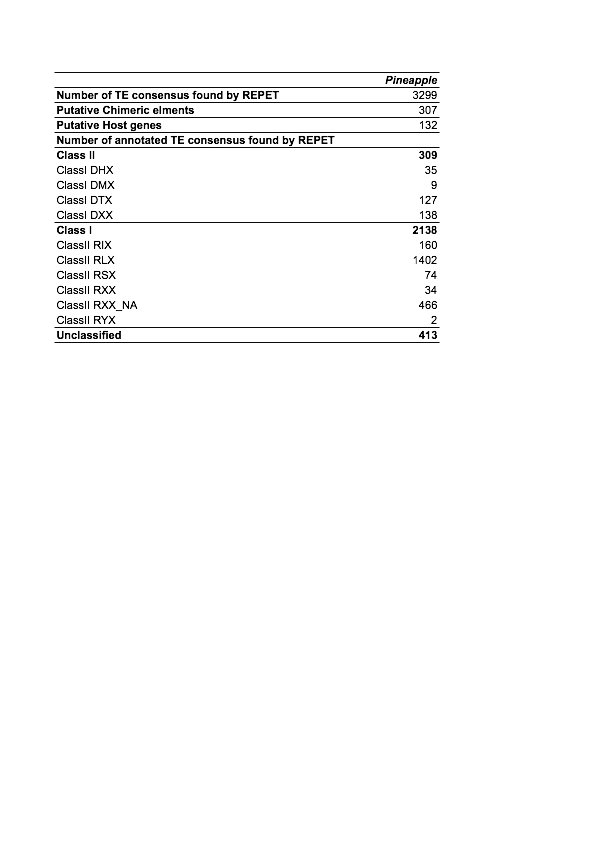
X LTR Retrotransposons, RSX SINE, RXX unclassified Retrotransposons, RXX_NA Non-autonomous Retrotransposons, RYX DIRS, Class II: DHX Helitron, DMX Maverick, DTX TIR Transposons, DXX MITE.
