# Supplementary material for: Inpactor, Integrated and Parallel Analyzer and Classifier of LTR Retrotransposons and Its Application for Pineapple LTR Retrotransposons Diversity and Dynamics
Source: Biology (Basel). 2018 May 25;7(2):32. doi: 10.3390/biology7020032 (PMC6022998; doi:10.3390/biology7020032)
Supplement: Supplementary file 1 [file biology-07-00032-s001.zip › Supplementary S11.pdf]

	LG1	LG2	LG3	LG4	LG5	LG6	LG7	LG8	LG9	LG10	LG11	LG12	LG13	LG14	LG15	LG16	LG17	LG18	LG19	LG20	LG21	LG22	LG23	LG24	LG25
**RLC-NO_FAMILY**	0,07	0,05	0,05	0,03	0,04	0,11	0,06	0,07	0,04	0,03	0,02	0,05	0,06	0,02	0,04	0,04	0,03	0,03	0,02	0,07	0,03	0,03	0,02	0,03	0,00
**RLC-ORYCO**	0,09	0,14	0,16	0,11	0,10	0,04	0,14	0,07	0,15	0,10	0,10	0,12	0,12	0,15	0,14	0,21	0,16	0,26	0,06	0,12	0,12	0,09	0,22	0,15	0,03
**RLC-RETROFIT**	0,33	0,32	0,38	0,27	0,34	0,38	0,33	0,35	0,35	0,16	0,49	0,31	0,45	0,32	0,34	0,20	0,24	0,45	0,22	0,41	0,18	0,30	0,29	0,20	0,17
**RLC-SIRE**	0,77	1,07	0,88	0,56	0,78	0,83	0,50	0,81	0,71	0,82	0,41	0,60	0,73	0,41	0,47	0,88	0,75	0,83	1,00	0,92	1,13	0,57	0,59	1,10	0,12
**RLC-TORK**	1,04	1,08	1,36	0,90	1,21	1,12	1,22	1,19	1,13	1,27	1,38	1,21	1,09	1,06	0,88	1,49	1,05	0,93	1,04	1,34	1,39	1,06	1,14	1,38	0,81
**RLG-CRM**	0,99	1,16	0,90	0,95	0,94	1,21	0,84	1,17	1,01	1,15	1,00	1,17	1,10	1,04	1,15	1,01	1,29	0,96	0,98	1,20	0,99	1,19	0,86	1,19	0,78
**RLG-DEL**	**15,53**	**14,48**	**13,70**	**11,69**	**12,15**	**14,57**	**14,61**	**11,68**	**12,06**	**13,80**	**14,51**	**11,62**	**15,28**	**12,39**	**13,41**	**14,34**	**11,48**	**13,04**	**15,47**	**14,45**	**13,56**	**13,63**	**13,48**	**18,65**	**4,74**
**RLG-GALADRIEL**	0,06	0,05	0,12	0,03	0,06	0,07	0,03	0,06	0,05	0,14	0,10	0,12	0,09	0,06	0,15	0,15	0,07	0,03	0,03	0,08	0,07	0,02	0,23	0,13	0,07
**RLG-REINA**	0,54	0,33	0,48	0,39	0,41	0,52	0,38	0,36	0,31	0,26	0,52	0,28	0,48	0,37	0,42	0,26	0,30	0,38	0,38	0,49	0,29	0,29	0,45	0,38	0,31
**RLG-TAT**	1,79	1,10	1,01	1,03	1,11	1,28	0,96	1,26	1,77	0,91	1,28	0,97	1,02	1,09	0,81	1,32	1,59	1,16	1,25	1,08	1,44	1,03	1,12	1,70	0,20
**RXX**	0,52	0,45	0,43	0,41	0,52	0,53	0,51	0,47	0,53	0,68	0,48	0,53	0,51	0,52	0,46	0,56	0,50	0,45	0,46	0,50	0,59	0,60	0,45	0,49	0,53
**TR-GAG**	6,93	6,25	6,64	5,79	6,62	7,27	6,22	6,12	6,67	6,67	7,01	7,10	7,42	6,70	7,12	7,47	6,57	6,76	6,86	7,02	6,55	6,38	6,91	7,78	2,94
**Total**	**28,66**	**26,48**	**26,11**	**22,16**	**24,28**	**27,93**	**25,80**	**23,61**	**24,78**	**25,99**	**27,30**	**24,08**	**28,35**	**24,13**	**25,39**	**27,93**	**24,03**	**25,28**	**27,77**	**27,68**	**26,34**	**25,19**	**25,76**	**33,18**	**10,70**
