# Supplementary material for: Inpactor, Integrated and Parallel Analyzer and Classifier of LTR Retrotransposons and Its Application for Pineapple LTR Retrotransposons Diversity and Dynamics
Source: Biology (Basel). 2018 May 25;7(2):32. doi: 10.3390/biology7020032 (PMC6022998; doi:10.3390/biology7020032)
Supplement: Supplementary file 1 [file biology-07-00032-s001.zip › Supplementary S1.docx]

######################## configuration file ###########################

input=/home/user/input

result_directory=/home/user/result_directory

verbose=true

clean=false

#this type can be LTR_STRUC, repet or fasta

input_type=LTR_STRUC

############################ Preprocessing ##############################

preprocessing=true

database=/home/user/DB/cores-database-wickercode.Lineage_Bianca.fa

########################### Classification ##############################

classification=true

# this tabfile isn't necessary if preprocessing is true

tabfile=/home/user/result_directory/step1/all_tabfiles.tab

80-80-80-rule=true

########################### Domain Extraction ###########################

extraction=true

RTdatabase=/home/user/result_directory/DB/RTcores-database-wickercode.Lineage_Bianca.fa

references=/home/user/result_directory/references.fasta

# this fastafile isn't necessary if classification is true

fastafile=/home/user/result_directory/step2/all_tabfiles.tab_ALL.RLC_RLG.FA

#filter RT size. For a lot of RT, is better RTlength > 200, for less RTlength > 180 or 150

RTlength=200

Blast_evalue=1e-4

########## Insertion Time analysis and Phylogenetic tree creation #######

insertion=true

substitution_rate=0.000000013

# this tabfile isn't necessary if classification is true

tabfileS4=/home/user/result_directory/step2/all_tabfiles.tab_ALL.RLC_RLG.TAB
