# Supplementary figures and images for: Inpactor, Integrated and Parallel Analyzer and Classifier of LTR Retrotransposons and Its Application for Pineapple LTR Retrotransposons Diversity and Dynamics
Source: Biology (Basel). 2018 May 25;7(2):32. doi: 10.3390/biology7020032 (PMC6022998; doi:10.3390/biology7020032)

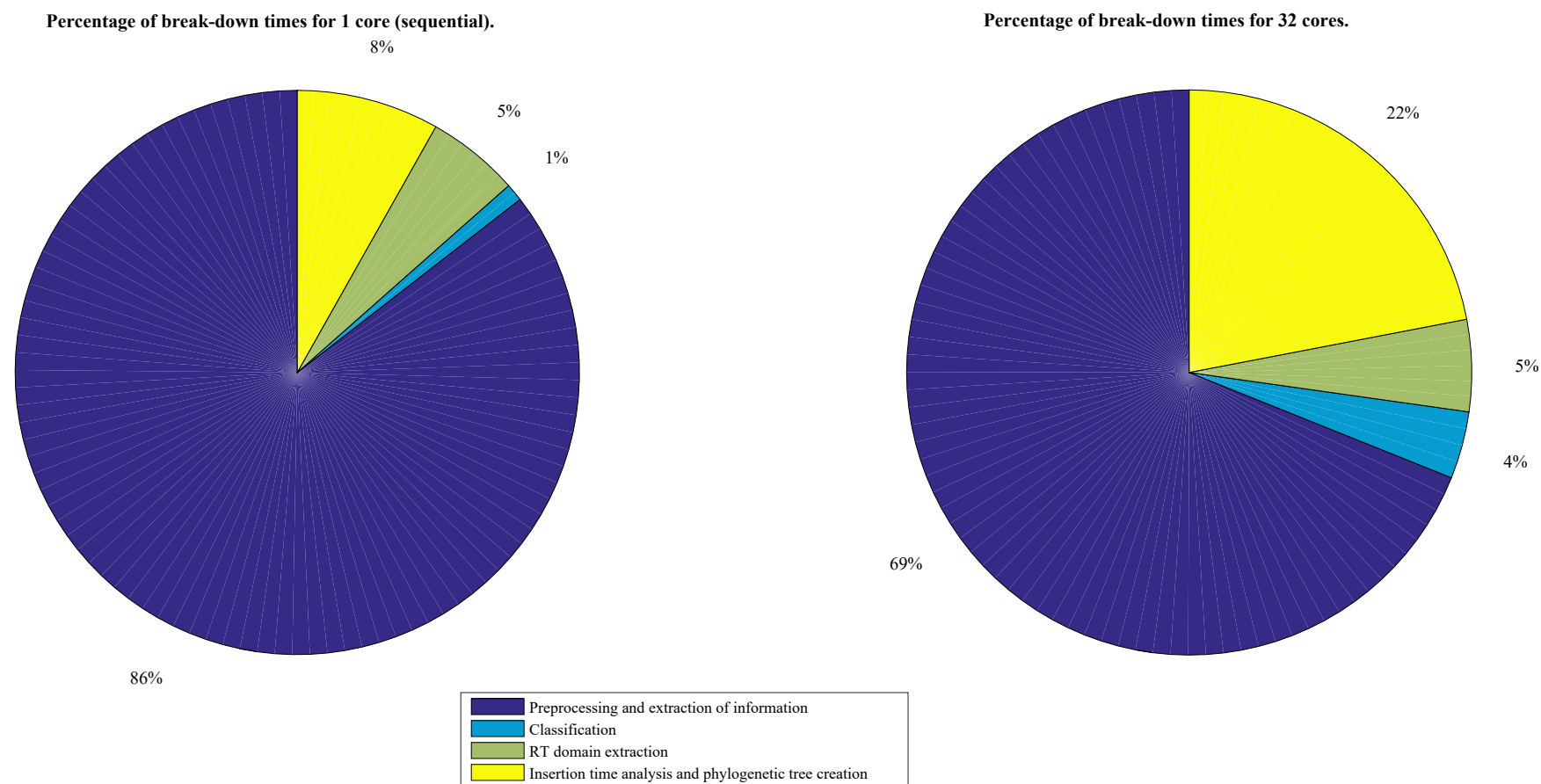

Supplement: Supplementary file 1 [file biology-07-00032-s001.zip › Supplementary S9.pdf]

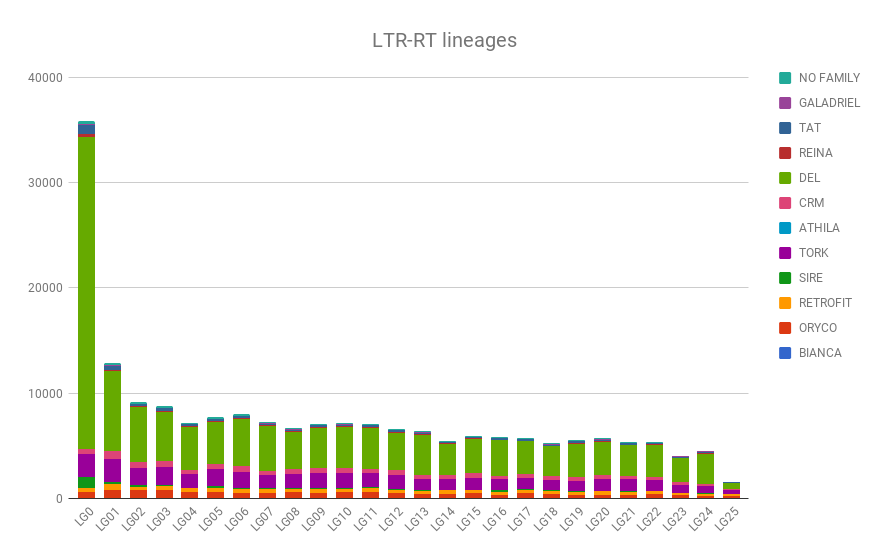

Supplement: Supplementary file 1 [file biology-07-00032-s001.zip › Supplementary S12.png]

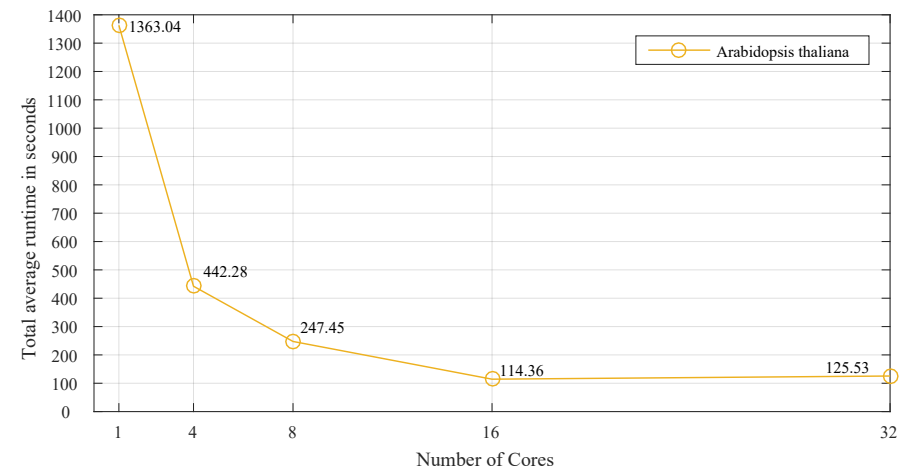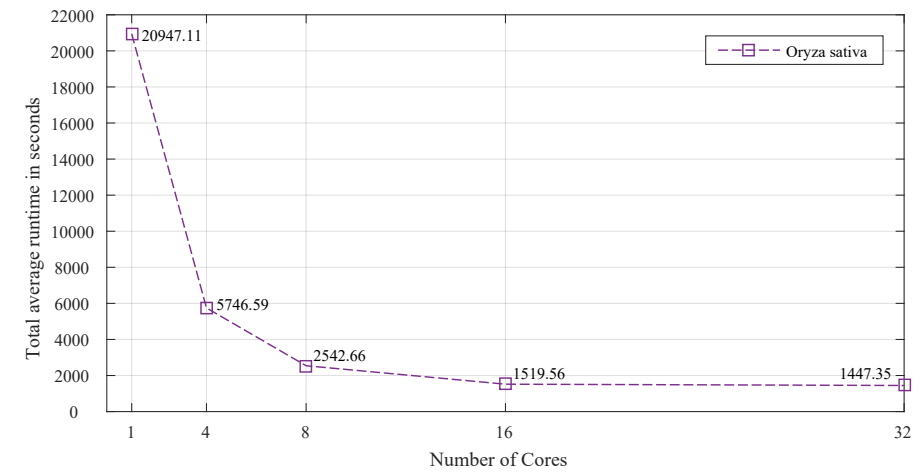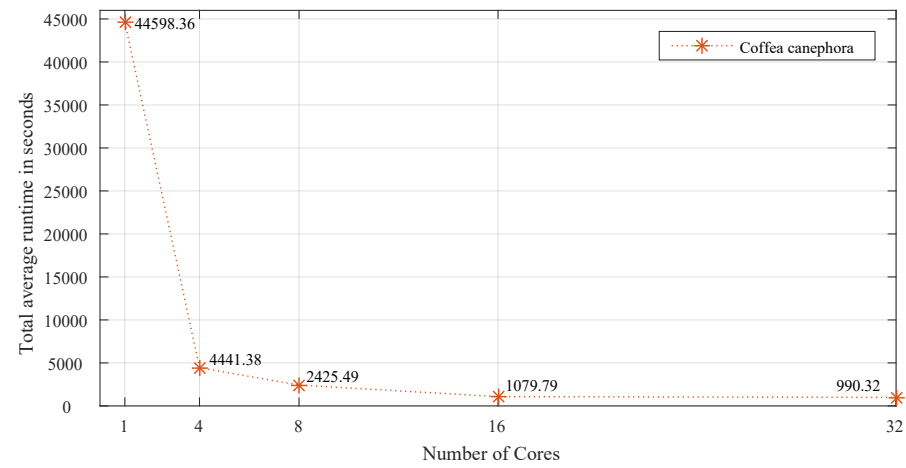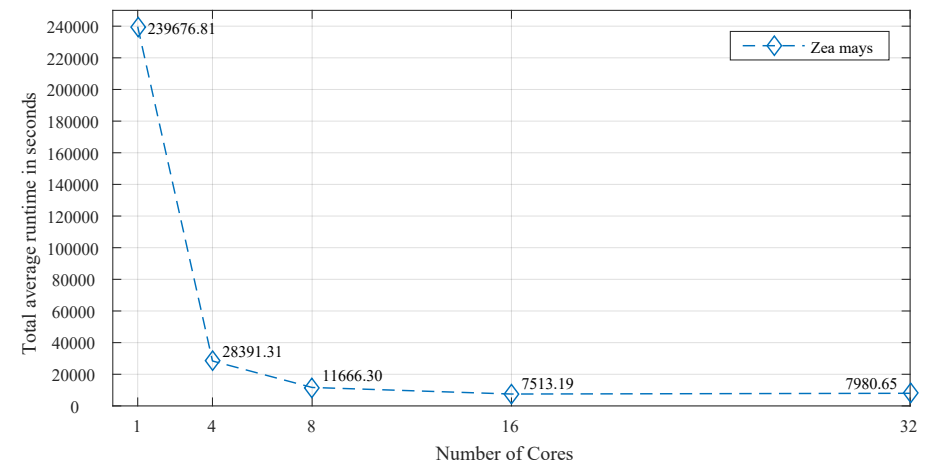

Supplement: Supplementary file 1 [file biology-07-00032-s001.zip › Supplementary S5.pdf]

Percentage of break-down times for 1 core (sequential).

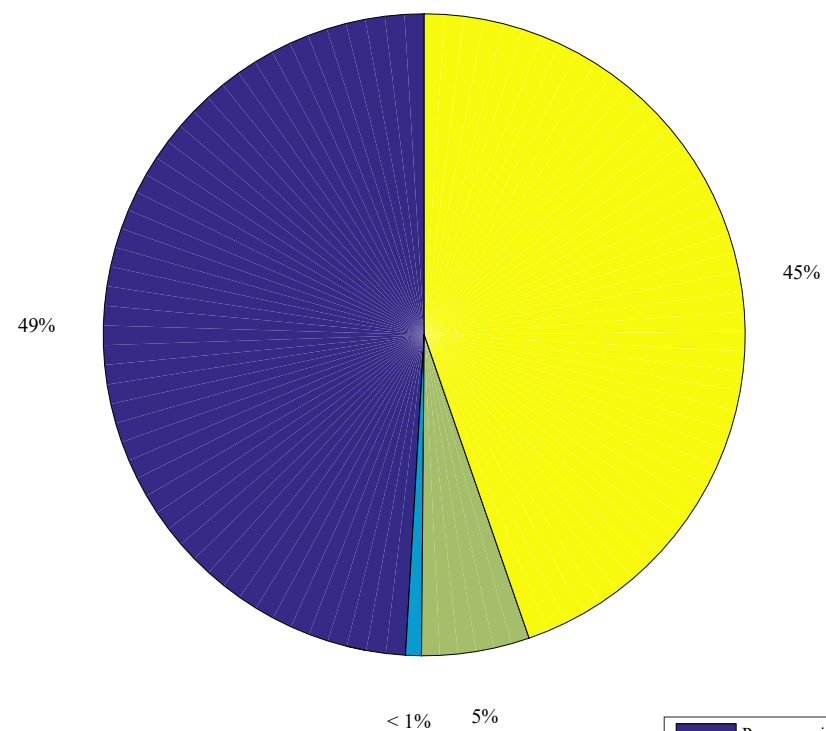

Percentage of break-down times for 32 cores.

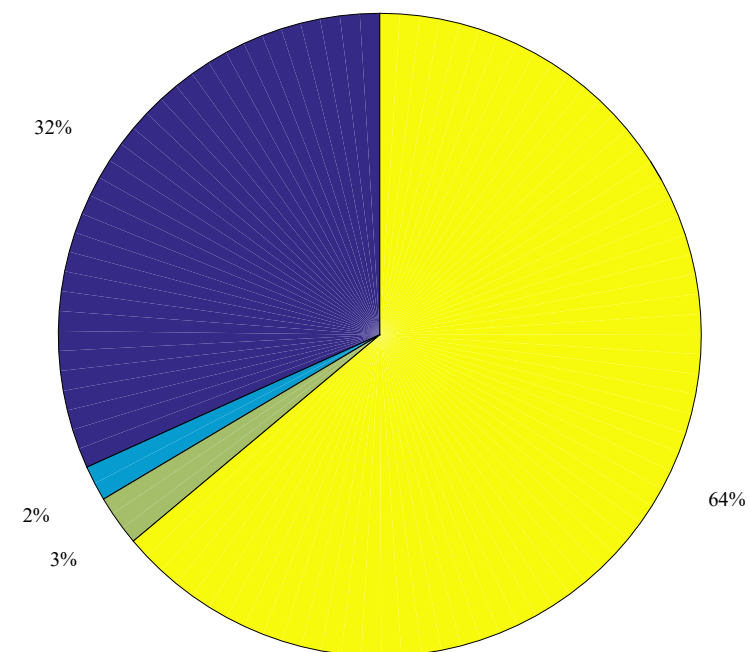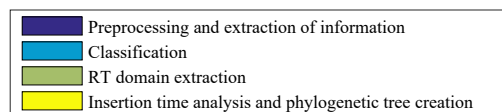

Supplement: Supplementary file 1 [file biology-07-00032-s001.zip › Supplementary S6.pdf]

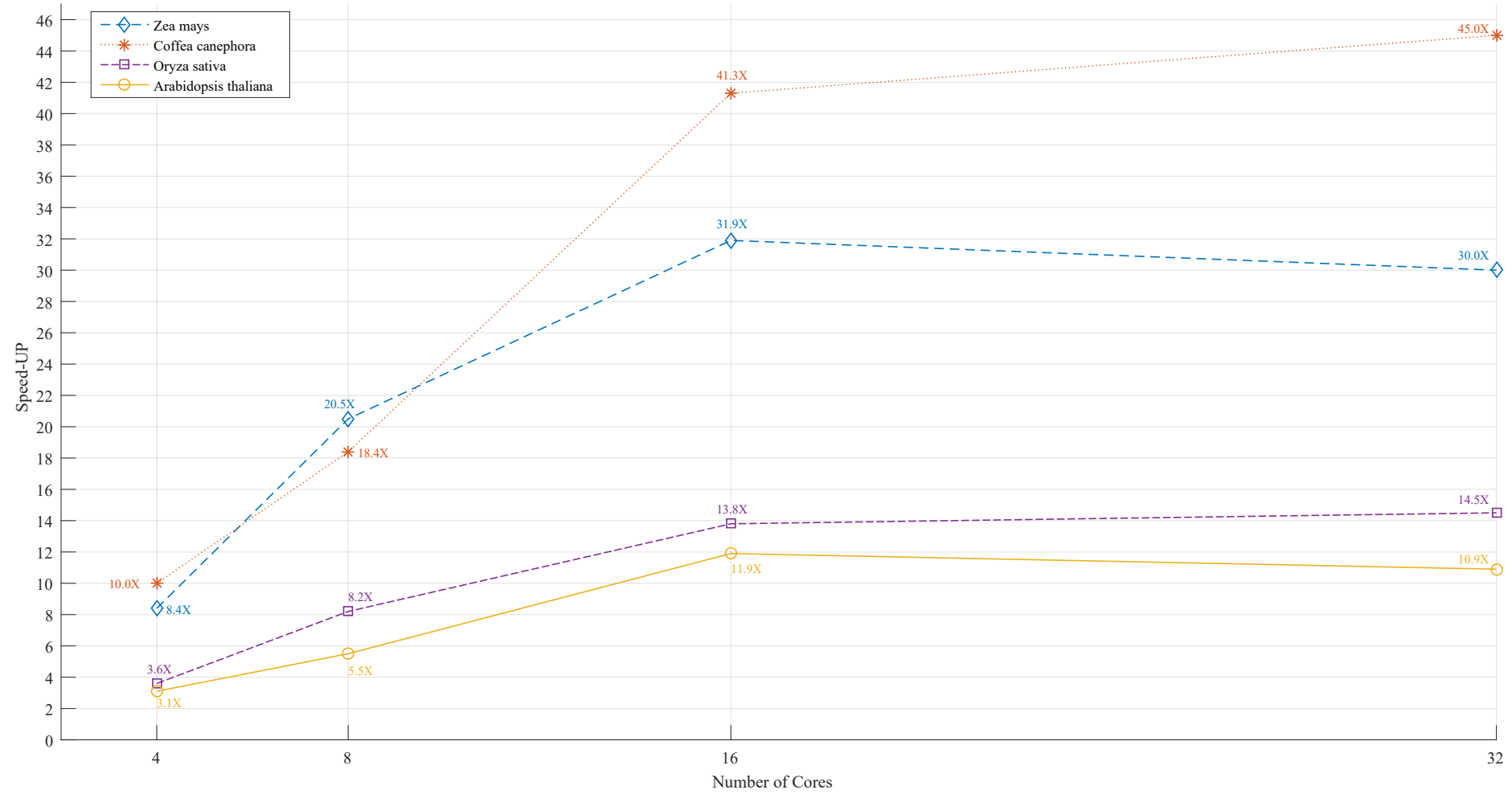

Supplement: Supplementary file 1 [file biology-07-00032-s001.zip › Supplementary S7.pdf]

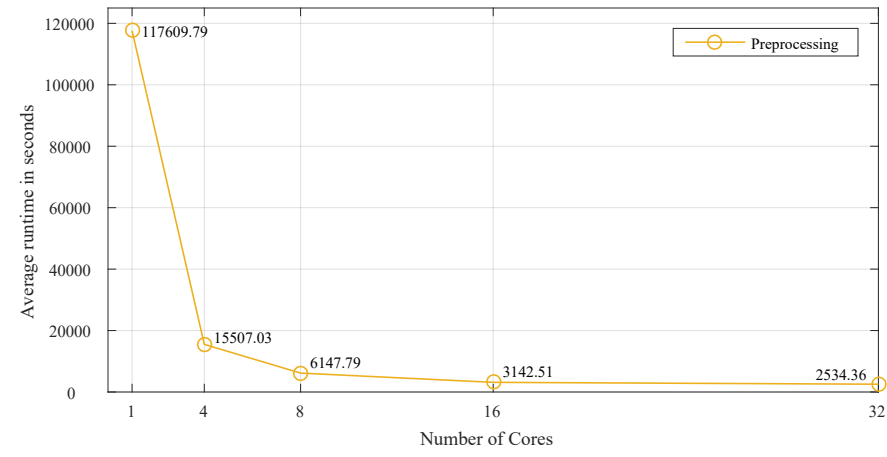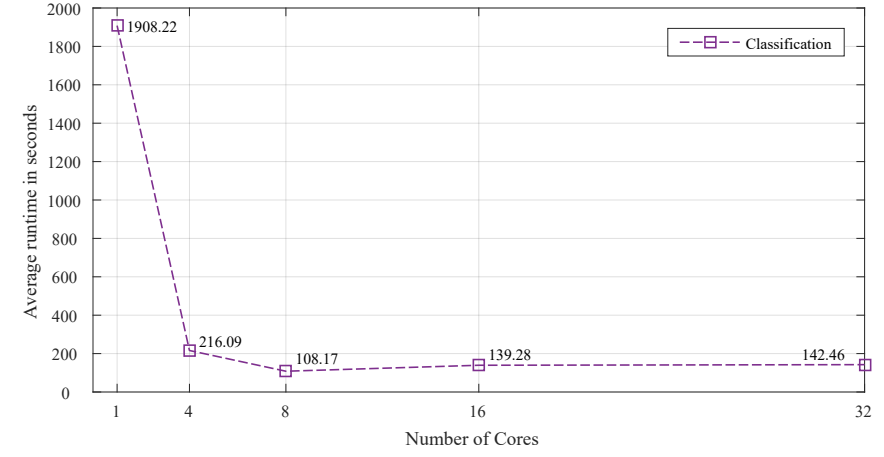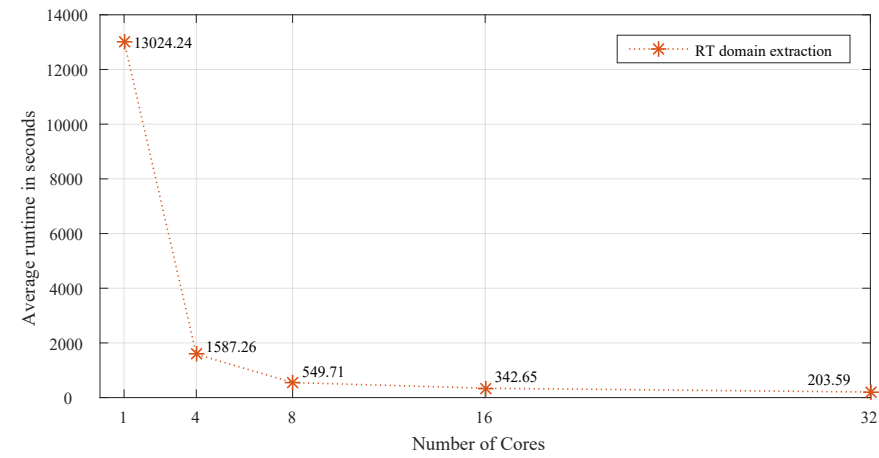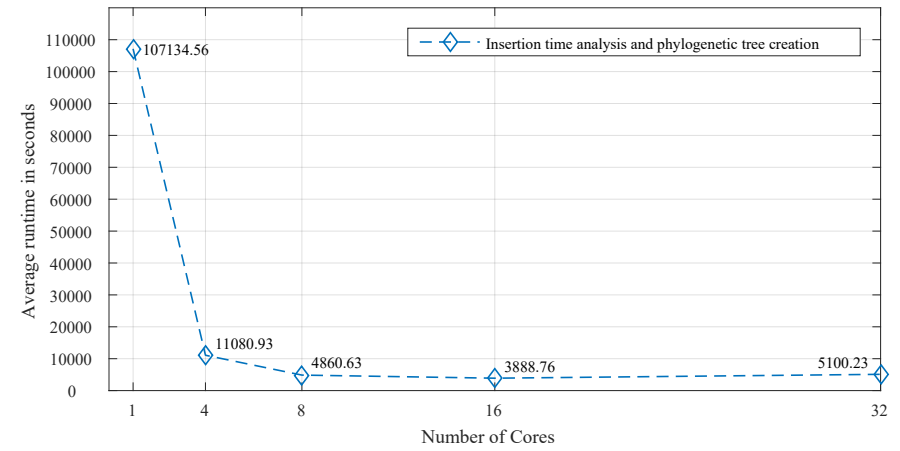

Supplement: Supplementary file 1 [file biology-07-00032-s001.zip › Supplementary S8.pdf]
